# Bisphenol A and bisphenol S both disrupt ovine granulosa cell steroidogenesis but through different molecular pathways

**DOI:** 10.1186/s13048-023-01114-4

**Published:** 2023-02-03

**Authors:** Ophélie Téteau, Anaïs Vitorino Carvalho, Pascal Papillier, Béatrice Mandon-Pépin, Luc Jouneau, Peggy Jarrier-Gaillard, Alice Desmarchais, Marie-Emilie Lebachelier de la Riviere, Claire Vignault, Virginie Maillard, Aurélien Binet, Svetlana Uzbekova, Sebastien Elis

**Affiliations:** 1 CNRS, IFCE, INRAE, Université de Tours, PRC, 37380 Nouzilly, France; 2 INRAE, BREED, Université Paris-Saclay, UVSQ, 78350 Jouy-en-Josas, France; 3 Service de Chirurgie Pédiatrique Viscérale, Urologique, Plastique Et Brûlés, CHRU de Tours, 37000 Tours, France

**Keywords:** Endocrine disruptors, Bisphenol, Granulosa cells, Steroidogenesis, Mechanisms of action, Ewe

## Abstract

**Background:**

Ovarian granulosa cells (GC) are essential for the development and maturation of a proper oocyte. GC are sensitive to endocrine disruptors, including bisphenol A (BPA) and its analogue bisphenol S (BPS), plasticisers present in everyday consumer products. BPA exhibits greater binding affinity for the membrane oestrogen receptor (GPER) than for the nuclear oestrogen receptors (ERα and ERβ). Here, we analysed the effects of BPA and BPS on the steroidogenesis of ovine GC in vitro, as well as their early mechanisms of action, the ovine being a relevant model to study human reproductive impairment. Disruption of GC steroidogenesis might alter oocyte quality and consequently fertility rate. In addition, we compared the effects of a specific GPER agonist (G-1) and antagonist (G-15) to those of BPA and BPS. Ewe GC were cultured with BPA or BPS (10 or 50 µM) or G-1 (1 µM) and/or G-15 (10 µM) for 48 h to study steroidogenesis.

**Results:**

Both BPA and BPS (10 µM) altered the secretion of progesterone, however, only BPS (10 µM) affected oestradiol secretion. RNA-seq was performed on GC after 1 h of culture with BPA or BPS (50 µM) or G-1 (10 µM), followed by real-time PCR analyses of differentially expressed genes after 12, 24 and 48 h of culture. The absence of induced GPER target genes showed that BPA and BPS did not activate GPER in GC after 1 h of treatment. These molecules exhibited mainly independent early mechanisms of action. Gene ontology analysis showed that after 1 h of treatment, BPA mainly disrupted the expression of the genes involved in metabolism and transcription, while BPS had a smaller effect and impaired cellular communications. BPA had a transient effect on the expression of *CHAC1* (NOTCH signalling and oxidative balance), *JUN* (linked to MAPK pathway), *NR4A1* (oestradiol secretion inhibition), *ARRDC4* (endocytose of GPCR) and *KLF10* (cell growth, differentiation and apoptosis), while expression changes were maintained over time for the genes *LSMEM1* (linked to MAPK pathway), *TXNIP* (oxidative stress) and *LIF* (cell cycle regulation) after 12 and 48 h, respectively.

**Conclusion:**

In conclusion, although they exhibited similar effects, BPA and BPS impaired different molecular pathways in GC in vitro. New investigations will be necessary to follow the temporal changes of these genes over time, as well as the biological processes involved.

**Supplementary Information:**

The online version contains supplementary material available at 10.1186/s13048-023-01114-4.

## Background

Within the ovarian follicle, granulosa cells (GC) develop around the oocyte. These cells delimit the antrum, filled with follicular fluid, which is composed of the secretory products of follicular cells and molecules provided by vascularisation (ions, nutrients, signalling molecules, metabolic precursors, growth factors and hormones). Thus, GC provide an optimal environment for the development and maturation of the oocyte. One of the main functions of GC is the synthesis and secretion of steroid hormones: progesterone and oestradiol [1, 2]. Nevertheless, numerous environmental factors can affect the steroidogenesis of GC and thus alter the quality of oocytes and ultimately female reproductive success [3, 4].

Among them, Bisphenol A (BPA), one of the plasticisers used in many daily consumer products, such as food packaging [5], paper products [6] and dental sealants [7], is now a recognised endocrine disruptor. Thus, in several countries, including Canada, France, and Switzerland, it has been banned from the food industry [8]. However, BPA was then replaced by structural analogues, essentially the Bisphenol S (BPS). The main source of exposure to bisphenols is through contaminated food [9], but also through skin contact [10] and inhalation of dust [11]. Indeed, both BPA and BPS have been detected in human body fluids: blood (7.6–8.2 nM), urine (2.6–8.4 nM) and follicular fluid (4.4–8.1 nM) [12–15]. BPA and BPS are linked closely to various diseases, such as obesity, diabetes, disruption of thyroid function and cardiovascular disease [16–20]. Both BPA and BPS are involved in male and female reproductive dysfunctions [21–23]. Bisphenol impairments occuring at several steps during both spermatogenesis and folliculogenesis, these deleterious effects could accumulate and contribute to decrease in fertility observed in human population and highlighted by the increase in the population undergoing ART. Indeed, in female mammals, numerous publications have highlighted how BPA and BPS alter mouse follicular development [24] as well as mouse, bovine and ovine oocyte and embryonic development [25–29]. Moreover, BPA and BPS impair steroidogenesis in rodent, porcine, bovine, human or ovine GC [3, 12, 30–38]. Nevertheless, steroid secretion by GC in the presence of bisphenols varies according to the conditions of the study (culture time, concentration, etc.) and the animal species.

Bisphenols are considered oestrogen mimetics due to their ability to bind nuclear oestrogen receptors, ERα and ERβ [39, 40]. The adverse effects of BPA may also be mediated by the membrane oestrogen receptor (GPER, also called GPR30) [41, 42]. GPER could produce rapid biological responses by activating several signalling pathways leading to intracellular Ca^2+^ mobilisation, cyclic adenosine monophosphate (cAMP) production, activation of various kinases and gene expression regulation. All these pathways regulate a wide variety of physiological processes (cell proliferation, metabolism, migration and secretion). In addition, the affinity of BPA for GPER is 50 times greater than for ERα [43, 44].

So far, the underlying mechanisms of action leading to alterations in GC functions in the presence of BPA or BPS remain poorly understood. The relevance of the ewe model has already been well described for studies on female reproduction [2, 45] and toxicological studies with BPA [46, 47]. We hypothesised that BPA and BPS alter the steroidogenesis of GC through GPER activation. To test this hypothesis, we performed both complementary pharmacological and transcriptomic analyses. We treated GC either after short treatment of elevated concentrations of BPA or BPS, and/or with the G1 agonist or G15 antagonist of GPER to see whether bisphenols or GPER activation / inhibition acted the same way on progesterone and oestradiol secretion. Then, we carried out RNA sequencing (RNA-seq) of BPA, BPS or G1 treated cells to investigate the early mechanisms of action of BPA and BPS after 1 h exposure in ovine GC in vitro and to compare the genes and corresponding biological processes affected by these molecules with genes regulated by GPER activation. Because we were mostly interested by primary targets of bisphenols, meaning regulations directly due to the binding of bisphenols to its putative ligands (including GPR30), we chose to perform a 1 h treatment. In addition, we studied the kinetics of the expression of eight differentially expressed genes (DEG) in GC after 12, 24 and 48 h of exposure. The concentration of 50 µM was used even if it is a high dose compared to environmental exposure. The point was to activate all potential pathways, even though depending on concentrations (and therefore affinity for receptors), different pathways could be activated. The treatment lasted only 1 h for RNAseq analysis in order to enable to investigate only the early steps and direct effects of the molecules.

## Results

### Pharmacological approach: effects of BPA, BPS and the GPER-specific agonist (G-1) and antagonist (G-15) on ovine GC steroidogenesis

The treatments (48 h) had no effect on cell viability regardless of the method used (Supplementary Figure S1B): Live/Dead staining (Supplementary Figure S1B.a), dehydrogenase activity assay (Supplementary Figure S1B.b) and lactate dehydrogenase activity assay in the medium (Supplementary Figure S1B.c).

**Fig. 1:**
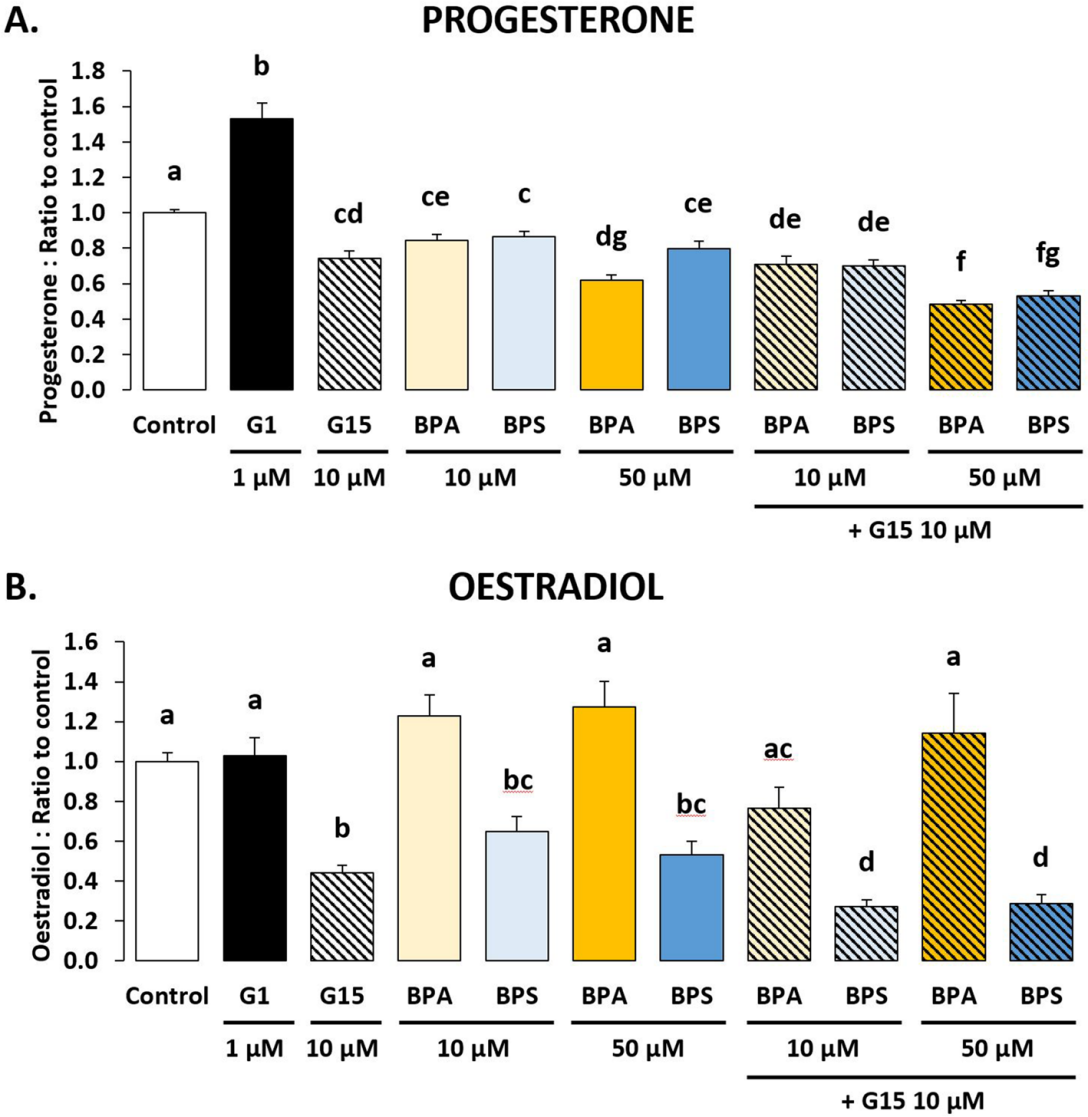
The effects of the GPER-specific agonist (G-1) or antagonist (G-15) and/or bisphenol A (BPA) and bisphenol (BPS) on ovine granulosa cells (GC) steroidogenesis. The progesterone (**A**) and oestradiol (**B**) concentrations were determined in culture medium after 48 h of culture in complemented serum-free McCoy’s 5A media in the presence or absence (control) of BPA or BPS at 10 or 50 μM, and/or G-15 at 10 µM or G-1 at 1 μM. The results are expressed as the mean ± standard error of the mean of 10 independent cultures. Each condition was performed in duplicate and normalised to the control condition of each culture experiment. Bars with different superscripts indicate a significant difference (*p* ≤ 0.05). The actual control values were 37.07 ± 4.68 ng progesterone/mg protein (**A**) and 52.04 ± 2.21 pg oestradiol/mg protein **B**

Progesterone secretion (Fig. 1A) was increased (*p* < 0.001) by 53% (58.22 ± 15.37 ng progesterone/mg protein) after G-1 treatment at 1 µM compared with the control (37.07 ± 4.68 ng progesterone/mg protein) (Supplementary Table 1). Conversely, G-15 at 10 μM decreased (*p* < 0.001) progesterone secretion (26.39 ± 2.92 ng progesterone/mg protein) by 26% compared with the control. Similarly, BPA and BPS also decreased (*p* < 0.001) progesterone secretion by 16% and 14%, respectively, at 10 µM (30.31 ± 2.95 and 30.33 ± 2.60 ng progesterone/mg protein, respectively), and by 38% and 20%, respectively, at 50 µM (22.59 ± 2.52 and 27.79 ± 2.52 ng progesterone/mg protein, respectively) compared with the control. G-15 at 10 µM impaired progesterone secretion of ovine GC similarly to BPA or BPS. When G-15 was combined with BPA or BPS at 10 µM, progesterone secretion was decreased (*p* < 0.001) by 29% and 30%, respectively (25.45 ± 2.84 and 24.34 ± 2.23 ng progesterone/mg protein, respectively) compared with the control, and similarly to G-15 alone. When G-15 was combined with BPA or BPS at 50 µM, progesterone secretion was further decreased (*p* < 0.001) by 52% and 47%, respectively (17.08 ± 1.70 and 18.71 ± 1.83 ng progesterone/mg protein, respectively) compared with the control. This was also the case when compared with G-15 alone: progesterone was reduced by 35% (*p* < 0.001) after BPA 50 µM treatment, while it was reduced by 29% (*p* < 0.001) after BPS 50 µM treatment. When G-15 was combined with BPA or BPS at 50 µM, progesterone secretion was reduced by 28% (*p* = 0.003) or 34% (*p* < 0.001) compared with BPA or BPS at 50 µM.

Oestradiol secretion (Fig. 1B) was not impacted significantly by G-1 at 1 µM or BPA at 10 or 50 µM (52.66 ± 7.78, 59.26 ± 10.36 and 54.67 ± 6.12 pg oestradiol/mg protein, respectively) compared with control (52.04 ± 5.21 pg oestradiol/mg protein) (Supplementary Table 1). G-15 at 10 μM decreased (*p* < 0.001) oestradiol secretion (23.02 ± 3.55 pg oestradiol/mg protein) by 56% compared with the control. BPS at 10 or 50 µM also decreased (*p* < 0.001) oestradiol secretion by 35% and 47%, respectively (32.90 ± 6.44 and 23.18 ± 2.87 pg oestradiol/mg protein, respectively) compared with the control. BPS at 10 or 50 µM alone impaired oestradiol secretion of ovine GC, similarly to G-15 at 10 µM. When G-15 was combined with BPS at 10 or 50 µM, oestradiol secretion was decreased (*p* = 0.006 and *p* = 0.019, respectively) by 73% and 71%, respectively (17.26 ± 3.09 and 16.68 ± 2.59 pg oestradiol/mg protein, respectively) compared with the control. This was also the case when compared with G-15: oestradiol was reduced by 38% (*p* = 0.006) after BPS 10 µM treatment, while it was reduced by 35% (*p* = 0.019) after BPS 50 µM treatment. When G-15 was combined with BPS at 10 or 50 µM, oestradiol secretion was reduced by 58% (*p* < 0.001) or 46% (*p* = 0.012) compared with BPS at 10 or 50 µM, respectively. On the other hand, the association of BPA at 10 or 50 µM with G-15 at 10 µM did not affect oestradiol secretion (32.25 ± 2.92 and 43.50 ± 4.91 pg oestradiol/mg protein, respectively) compared with the control.

### Transcriptomic approach: effects of BPA, BPS and the GPER-specific agonist (G-1) on the ovine GC transcriptome

No effect on cell viability was observed after 1 h of treatment with G1 10 μM, BPA 50 µM or BPS 50 μM, using two complementary methods (Supplementary Figure S1A): Live/Dead staining (Supplementary Figure S1A.a) and the dehydrogenase activity assay (Supplementary Figure S1A.b).

**Fig. 2:**
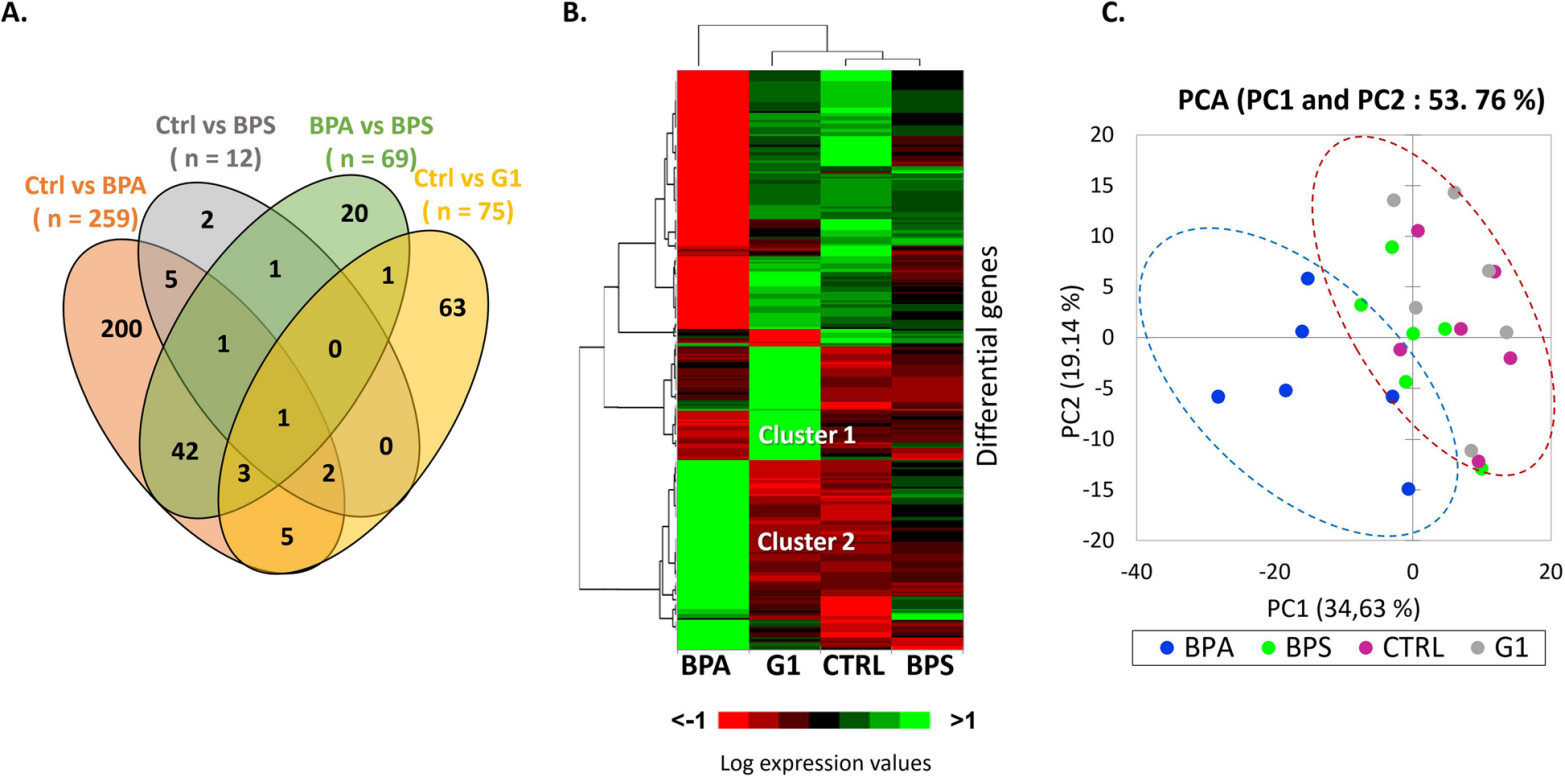
Differentially expressed genes (DEG) in ovine granulosa cells (GC) treated with bisphenol A (BPA), bisphenol S (BPS) or the GPER-specific agonist (G-1). After 1 h culture in complemented serum-free McCoy’s 5A media in the presence or absence (control) of BPA or BPS at 50 μM, or G-1 at 10 μM, six biological replicates were analysed with RNA-sequencing, and DEG were identified (padj ≤ 0.05). The Venn diagram (**A**) shows common and specific DEG from four comparisons: control versus BPA, control versus BPS, BPA versus BPS, and Control versus G-1. The heatmap (**B**) shows hierarchical clustering of DEG. The ordered list of heatmap genes have been annotated in Supplementary Table S3. Principle component analysis of gene expression in GC treated or not with BPA, BPS or G1 performed on expression values of 24 differential genes **C**

To investigate the early mechanisms of action of BPA and BPS on the steroidogenesis of ovine GC, 24 ovine GC samples corresponding to four experimental conditions (control, 50 µM BPA, 50 µM BPS and 10 µM G-1) with six biological replicates for each condition were used for RNA-seq. The analysis generated an average of 23.7 ± 0.5 million reads per sample (Supplementary Table 2). Around 73.7% ± 0.2% of the reads were aligned uniquely with the sheep reference genome. This analysis was performed by using the genetic profiles of 14,883 genes generated by the RNA-seq experiment. Among them, 346 DEG were detected in the four comparisons: control versus BPA, control versus BPS, BPA versus BPS and control versus G1, with 259, 12, 69 and 75 DEG, respectively (Fig. 2A and Supplementary Table 3). Only three DEG, *SREBF1*, *SPRY2* and *TXNIP*, were common between the control versus BPA, control versus BPS and control versus G-1 comparisons. The control versus BPA and control versus G-1 comparisons revealed 11 common DEG: *TXNIP*, *JUN*, *NR4A1*, *SPRY2*, *C15orf39*, *ENSOARG00000018728 novel gene*, *TUBB*, *PPP1R3C*, *GADD45B*, *SREBF1* and *STAR*. However, the majority of DEG induced by BPA (200/259, 77%) and G-1 (69/75, 92%) were specific modifications, not found in other comparisons. Moreover, nine DEG (*TXNIP*, *ENSOARG00000022521 novel gene*, *SPRY2*, *ENSOARG00000024133 novel gene*, *FAM217A*, *ANKRD1*, *DDIT4*, *MAPS2*, *SREBF1*) were common between the control versus BPA and control versus BPS comparisons. Thus, the majority of DEG found in the control versus BPS comparison (9/12, 75%) were with the same DEG found in the control versus BPA comparison (Fig. 2A). A hierarchical clustering showed that BPA modified the expression of the genes in an opposite direction compared with G1. Indeed, cluster 1 regrouped the genes, which were upregulated by BPA, whereas cluster 2 showed the genes that were upregulated by G-1 (Supplementary Table 3 and Supplementary Table 4). In addition, after 1 h of treatment, BPS 50 μM only mildly modified the transcriptomic profile of the ovine GC compared with the control (Fig. 2B and Supplementary Table 4), as confirmed by a Principal Component Analysis (Fig. 2C).

These results suggest that different genes were affected by BPA or BPS treatment; moreover, treatment with G-1, the specific agonist of GPER, affected a different set of genes. Therefore, the following analyses focused on the 283 DEG identified from three differential analyses: control versus BPA, control versus BPS and BPA versus BPS (Supplementary Table 3). From these DEG, 150 genes were downregulated by BPA (*ARRDC4*, *KLF10*, *LIF*, *KLF11*, *EGR3*, *KLF9*, *ENC1*, *NOTCH1*, and others) whereas 109 genes were upregulated by this molecule (*CHAC1*, *JUN*, *LSMEM1*, *NR4A1*, and others). Regarding BPS, only four genes were downregulated (*RHOB*, *ANKRD1, SPRY2* and *TXNIP*) and eight were upregulated (*RASL11B*, *PDK4*, *SREBF1*, *MASP2*, *DDIT4*, *FAM217A*, *ENSOARG00000024133 novel gene* and *ENSOARG00000022521 novel gene*) compared with the control. In addition, *TXNIP* and *SPRY2* were downregulated and six genes were upregulated (*FAM217A*, *SREBF1*, *DDIT4*, *MAPS2*, *ENSOARG00000024133 novel gene*, *ENSOARG00000022521 novel gene*) by both BPA and BPS compared with the control.

#### Effects of BPA and BPS on biological processes

**Fig. 3:**
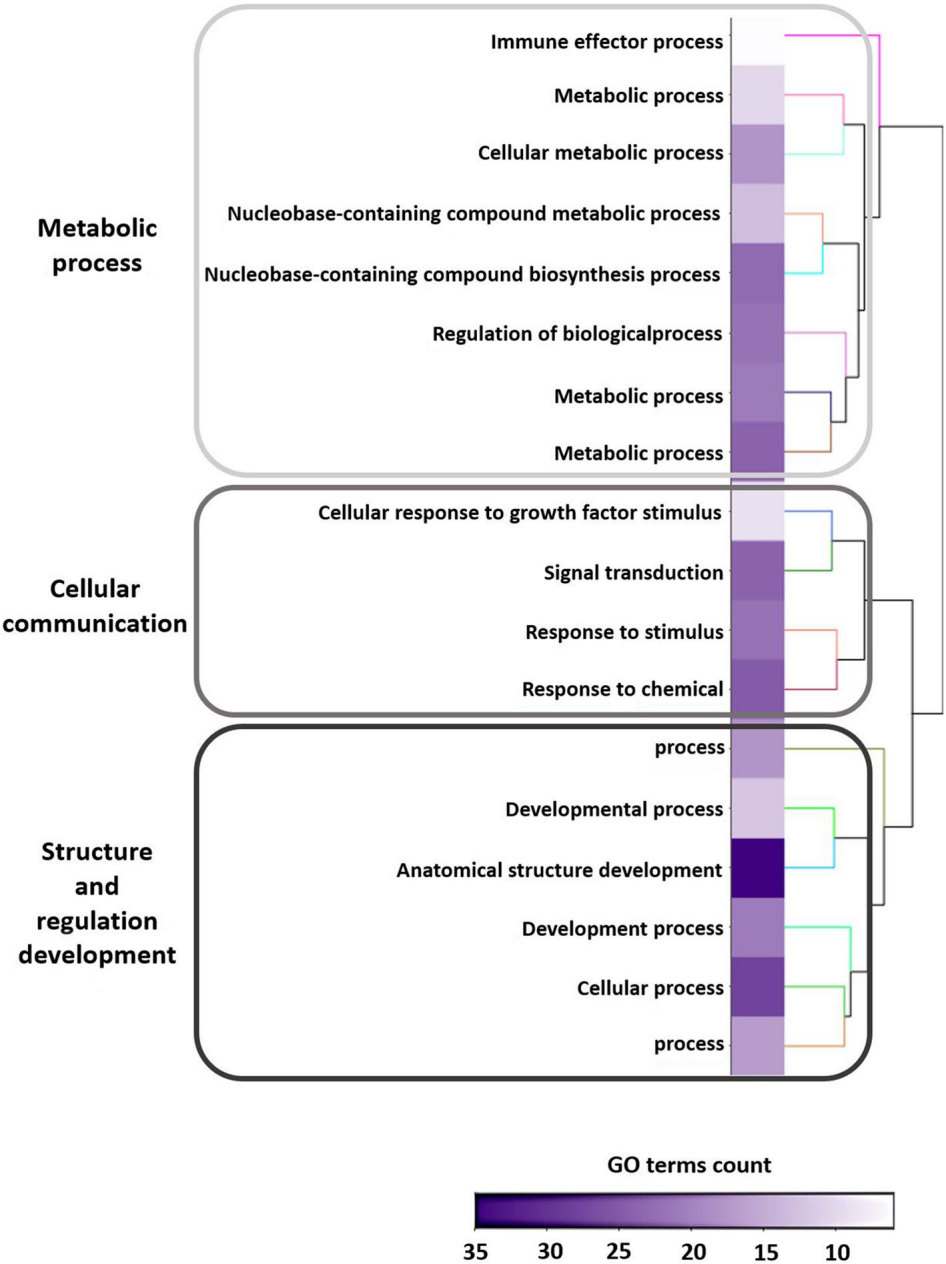
Functional analysis of differentially expressed genes (DEG) from RNA-sequencing of ovine granulosa cells (GC). After 1 h of treatment in the presence or absence (control) of bisphenol A (BPA) or bisphenol S (BPS) at 50 μM, GC (six replicates per condition) were analysed with RNA-sequencing to obtain a list of DEG (*p* ≤ 0.05). The global clustering heatmap plot of functional sets of gene ontology (GO; *p* ≤ 0.01) terms was obtained by using ViSEAGO. From left to right are: the major processes, the cluster name, a heatmap of GO term counts from functional enrichment tests and a dendrogram based on Wang’s semantic similarity distance and Ward’s clustering criterion

A functional analysis based on GO analysis was performed with the ViSEAGO R package [48] by hierarchical clustering of enriched GO terms. There were 348 enriched GO terms among the three lists of DEG (Supplementary Fig. 2 and Supplementary Table 5). These terms were grouped into 18 clusters that could be further categorised into three major processes: metabolic processes (8 clusters and 137 GO terms), cellular communication (4 clusters and 80 GO terms) and structure and regulation of development (6 clusters and 131 GO terms) (Fig. 3 and Supplementary Table 5).

The top 20 GO terms specifically altered by BPA correspond to three biological processes: metabolic processes (10 GO terms and 76/259 [29%] BPA DEG), transcription regulation (9 GO terms and 53/259 [20%] BPA DEG) and structure development (1 GO term and 25/259 [10%] BPA DEG). Regarding BPS, two others biological processes were impaired: cellular communication (18 GO terms) and cellular process (2 GO terms), representing 75% of BPS DEG (Fig. 2B). These five biological processes were also highlighted in the BPA versus BPS comparison. However, 18/20 GO (90%) corresponded to the effect of BPA while only 2/20 GO (10%) corresponded to the effect of BPS (Supplementary Table 6).

#### Interactions of DEG regulated by BPA

STRING analysis [49] was performed on 214 out of 259 (83%) BPA-regulated DEG to investigate the potential interaction network of the corresponding proteins (Supplementary Figure S3). Of note, the remaining DEG have not yet been annotated for the *O. aries* genome. This analysis highlighted the presence of several proteins involved in metabolic pathways (NDL4, MVD, SQLE, MAT2A, CYTB, ODC1, SETMAR, SGMS2, CYP2J, PIGH, DHCR24 and CHAC1) or in mitogen-activated protein kinase (MAPK) signalling pathways (SRF, ATF4, IL1A, MAP3K14, GADD45A, NR4A1 and GADD45B). These results confirm that metabolism is the primary biological process altered by BPA, therefore corroborating the GO analysis of DEG at the transcript level (Fig. 3). Using 12 DEG regulated by BPS, no common signalling pathways were identified.

#### Effects of BPA and BPS on the expression of most DEG after 12, 24 and 48 h

**Fig. 4:**
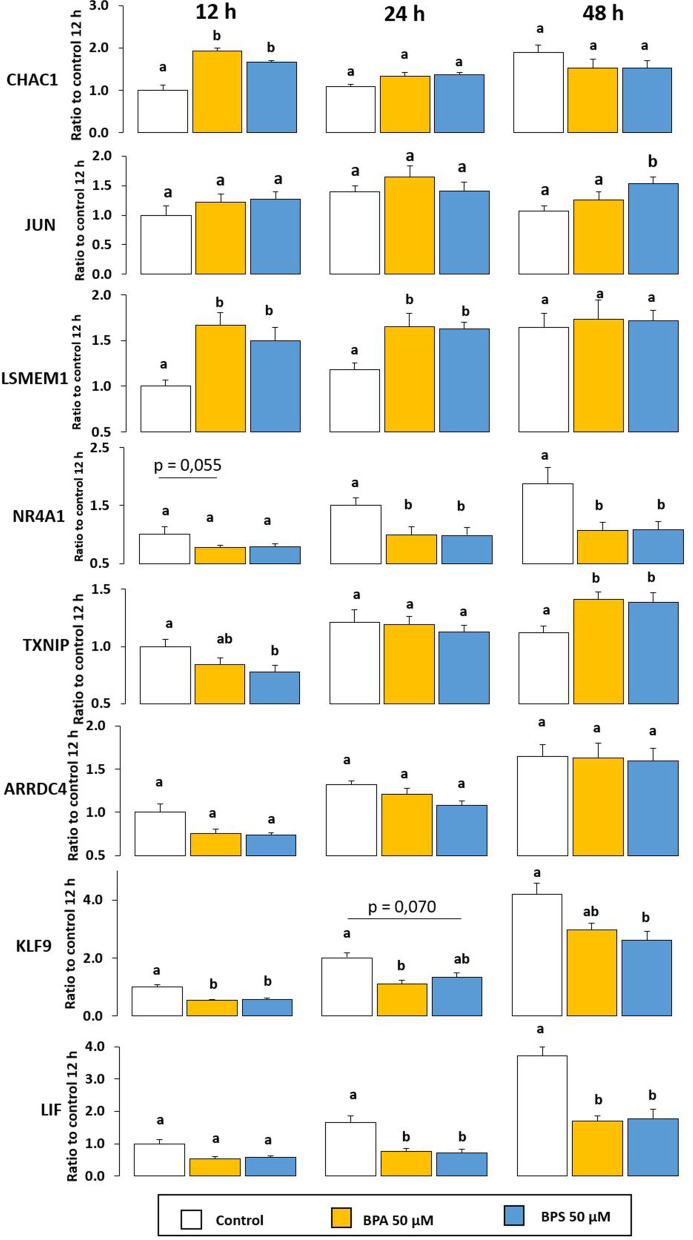
The effects of the GPER-specific antagonist (G-15) and/or bisphenol A (BPA) and bisphenol S (BPS) on gene expression of ovine granulosa cells (GC), according to changes over time. The expression of eight genes (*CHAC1*, *JUN*, *LSMEM1*, *NR4A1*, *TXNIP*, *ARRDC4*, *KLF9* and *LIF*) were assessed in ovine GC. Gene expression was determined after 12, 24 and 48 h of culture in complemented serum-free McCoy’s 5A media in the presence or absence (control) of BPA or BPS at 50 μM. Total messenger RNA (mRNA) was extracted and reverse transcribed from ovine GC, then real-time polymerase chain reaction (qPCR) was performed. To normalise gene expression, the geometric mean of two housekeeping genes (β-actin [ACTB] and ribosomal protein L19 [RPL19]) was used. The results are expressed as the mean ± standard error of the mean of eight independent cultures and normalised to the mean of the control condition at 12 h. Statistical analysis was performed between the conditions of a same time (12, 24 or 48 h). Bars with different superscripts indicate a significant difference (*p* ≤ 0.05)

Among the list of 283 DEG, obtained from the analysis of RNA-seq, the four most upregulated genes (*CHAC1*, *JUN*, *LSMEM1* and *NR4A1*) and the four most downregulated genes (*TXNIP*, *ARRDC4*, *KLF10* and *LIF*) in BPA compared to control were selected (Supplementary Table 3), with a fold-change of 1.5 (|FC|> 1.5), an adjusted *p*-value ≤ 0.05 and a sufficient number of reads (> 118) to perform qPCR. qPCR analyses of expression of these eight genes were performed in GC 12, 24 or 48 h after treatment with BPA or BPS 50 µM (Fig. 4 and Supplementary Table 7).

Similarly to 1 h of treatment, after 12 h of treatment, *CHAC1* expression was significantly increased after both BPA (+ 93%, *p* < 0.001) and BPS (+ 66%, *p* < 0.001) treatment compared with the control. After 24 h and 48 h, no difference in *CHAC1* expression was observed among the treatments (Fig. 4). Nevertheless, while in control condition *CHAC1* expression increased as the culture time increased, the same pattern was not observed after BPA or BPS treatment (Supplementary Table 7).

After 12, 24 or 48 h of treatment, no significant effect on *JUN* expression was observed in any condition, except BPS that increased *JUN* expression after 48 h (+ 43%, *p* = 0.002) compared with the control.

*LSMEM1* expression was still increased after 12 h and 24 h treatment with BPA 50 μM (+ 66%, *p* < 0.001 and + 40%, *p* = 0.005, respectively) or BPS 50 μM (+ 50%, *p* = 0.007 and + 38%, *p* < 0.001, respectively) compared with the control. There was no difference in *LSMEM1* gene expression after 48 h of treatment (Fig. 4).

While *NR4A1* gene expression was upregulated after 1 h of BPA 50 µM treatment compared with control (Supplementary Table 3), there was no difference after 12 h of treatment in any condition. *NR4A1* gene expression was reduced with BPA (-33%, *p* = 0.046) and BPS 50 µM (-33%, *p* = 0.030),after 24 h treatment. Similarly, *NR4A1* gene expression was reduced with BPA 50 µM (-43%, *p* = 0.013) and BPS (-42%, *p* = 0.013).

Thus, among *CHAC1*, *JUN*, *LSMEM1* and *NR4A1* – all upregulated after 1 h of BPA 50 µM treatment according to RNseq analysis (Supplementary Table 3) – only *LSMEM1* expression was still increased after 12 h and 24 h of treatment according to qPCR analysis (Fig. 4).

*TXNIP* was downregulated after 1 h of BPA 50 µM (Supplementary Table 3). This downregulation was also observed after 12 h of BPS 50 µM treatment (-22%, *p* = 0.014) (Fig. 4). No effect was observed after 24 h culture. After 48 h culture, both BPA 50 µM (+ 26%, *p* = 0.018) and BPS 50 µM (+ 24%, *p* = 0.002) increased *TXNIP* expression compared with the control. An increase of *TXNIP* expression is also observed over time for both BPA and BPS 50 µM, but not for the control (Supplementary Table 7).

*ARRDC4* gene expression was not reduced anymore after 12 h and 24 h of BPA or BPS 50 µM treatments compared with control. No difference was observed in *ARRDC4* gene expression after 48 h, regardless of the treatment (Fig. 4). *ARRDC4* gene expression expression is also increased over time in all conditions (Supplementary Table 7).

*KLF10* expression did not change after 12 h, 24 h or 48 h of treatment compared to the control. Two other members of the *KLF* family, *KLF9* and *KLF11*, were also BPA-induced DEG. After 12 and 24 h of treatment, *KLF9* expression was reduced after treatment with BPA 50 µM (-46%, *p* < 0.001, and -44%, *p* < 0.001, respectively) and BPS 50 µM (-43%, *p* < 0.001, and -33%, *p* = 0.070, respectively) compared with the control. After 48 h, KLF9 expression was still reduced after BPS 50 µM treatment (-38%, *p* = 0.008). *KLF9* expression increased over time (Supplementary Table 7). *KLF11* expression did not differ between conditions at any time (Supplementary Table 7).

Finally, *LIF* expression was still reduced after 24 h (-54%, *p *< 0.001) and 48 h (-54%, *p* < 0.001) BPA 50 µM treatment compared with the control. The same effect was observed after 24 h (-57%, *p* < 0.001) and 48 h (-53%, *p* < 0.001) BPS 50 µM treatment (Fig. 4). *LIF* expression increased over time (Supplementary Table 7).

Among the four genes (*TXNIP*, *ARRDC4*, *KLF10* and *LIF*) that were downregulated after 1 h of BPA 50 µM treatment according to RNAseq analysis (Supplementary Table 3), only *TXNIP* and *LIF* were also downregulated after either 12 h or 24 h or 48 h according to qPCR analysis (Fig. 4).

Two other downregulated genes after BPA treatment compared to control were also analysed by qPCR (*EGR3* and *NOTCH1*). No change in *EGR3* expression was observed between conditions. *NOTCH1* expression was decreased after 1 h of 50 µM BPA treatment (FC = 0.729, *p* = 0.036) compared with the control (Supplementary Table 3). On the contrary, after 12 h of treatment with 50 µM BPA or BPS, *NOTCH1* expression was increased (+ 52%, *p* < 0.001, and + 64%, *p* < 0.001, respectively).

## Discussion

This study aimed to highlight the early mechanisms of action of BPA and BPS on ovine GC. Both BPA and BPS impaired progesterone secretion, while only BPS inhibited oestradiol secretion. Thus, BPA and BPS exerted partially different effects on ovine GC steroidogenesis. To our knowledge, our exploratory study is the first to analyse BPA or BPS early mechanisms of action (at 1 h) on a model of ovine GC using RNA-seq. The comparison with the specific agonist of GPER (G-1) revealed that neither BPA nor BPS acted mainly through this receptor in our conditions. In addition, BPA and BPS seems to act through independent mechanisms. BPA altered genes mainly affecting cell metabolic process, while BPS only mildly altered gene expression and affected mostly the cell communication process. Finally, for the majority of analysed genes, BPA only affected their expression transiently.

### BPA and BPS impaired ovine GC steroidogenesis

In the present study, 48-h exposure to BPA or BPS decreased progesterone secretion by ovine GC in vitro. These results are in agreement with our previous study [33], although this inhibitory effect was observed at a different BPA concentration. Regarding the oestradiol level, BPA had no effect, whereas BPS caused a significant decrease in oestradiol secretion. First, the difference in BPA and BPS effects on oestradiol secretion suggests that they act, at least partly, by independent mechanisms, although they are structural analogues. Moreover, studies on bisphenol effects on steroidogenesis in GC of other species have also shown differences in the intensity and direction of variation of the effects. For example, contradictory results on oestradiol secretion by porcine GC in the presence of 10 µM BPA in vitro have been reported, with either an increase [35] or a decrease [34, 50] in the oestradiol level between treated and control groups. Other studies have reported either a decrease in progesterone secretion from rat GC [51], or its significant increase [52] after BPA treatment, depending on the concentration used. Moreover, these results are not in line with our previous study in which oestradiol secretion increased after treatment of GC culture with BPA or BPS treatment for 48 h [33]. Even though the same GC culture protocol was used and despite the large number of ewe ovaries (approximately 1,000) collected from slaughterhouses for follicle puncture, the inter-individual variability was not controlled. Ewes were of different ages, breeds, metabolic status and period of the year, among other factors. All these parameters can affect the reproductive system [53–56], especially the oestrus period [57], and, consequently, the effects of endocrine disruptors on the GC from these animals. Moreover, in a previous work, a steroidome assay was performed in an in vivo study, where the ewes with contrasted metabolic status (restricted versus well-fed) were chronically exposed to BPS via the diet. The effect of BPS on the pre-ovulatory follicular fluid steroidome varied according to the metabolic status of the animal. Indeed, a stimulatory effect of BPS on the oestradiol level was observed only in well-fed ewes [58]. From this perspective, it could be assumed that the difference in sensitivity to bisphenols and variation in steroidogenesis of ovine GC could be influenced by either the metabolic status of the ewes – the donors of GC and/or the oestrus season. These findings highlight the importance of taking into account animal metabolic status to investigate the effects of environmental factors or specifically the effects of endocrine disruptors on derived cells. Therefore, further studies performed on the cells originating from the animals with identified metabolic status, age and breed, among other factors, are necessary to decipher precisely the effects of BPA and BPS on GC steroidogenesis.

While the GPER agonist G-1 increased progesterone secretion, the GPER antagonist G-15 led to a significant decrease, comparable to BPA or BPS. However, when G-15 was combined with 50 µM BPA or BPS, the decrease in progesterone secretion was greater compared with G-15, BPA or BPS alone, therefore suggesting a cumulative effect. Such a cumulative effect of G-15 and BPS was also observed on oestradiol secretion, while BPA had no effect. Thus, contrary to our initial hypothesis, BPA and BPS at these concentrations does not seem to act mainly through GPER to modify the progesterone or oestradiol secretion of ovine GC. These results also showed that BPA and BPS do not share the same mechanisms of action.

Of note, to our knowledge, no study has evaluated the involvement of GPER on the effects of bisphenols on GC steroidogenesis. Nevertheless, BPA stimulated the proliferation of ovarian cells via GPER in the OVOCAR-3 and KGN cancerous GC lines [42], while BPA and BPS showed only a mild inhibitory effect on primary ovine GC proliferation [33]. However, several studies have shown the involvement of GPER in different cancers [59–61], potentially explaining this difference in BPA mechanisms of action in primary GC versus cancerous GC lines.

### BPA and BPS do not act predominantly through GPER

The RNA-seq analysis of GC cultured in the presence of the GPER-specific agonist G-1 showed a specific gene pattern, different from the patterns observed after either BPA or BPS treatment. The characterisation of GPER is recent, and its involvement in oestrogenic responses in reproductive organs remains controversial [62]. Environmental oestrogens, such as bisphenols, could activate several receptors in parallel (ERα, ERβ, GPER and others). Their effects depend on their concentration and affinity for each receptor, a factor that can partially explain their non-linear effect [63]. Only a few DEG were common between treatment with BPA or BPS and G1, including *STAR*, a cytoplasmic cholesterol transporter involved in steroidogenesis [3]; *SREBF1*, a transcription factor involved in the regulation of the biosynthesis of fatty acids and cholesterol [64]; and *SPRY2*, a negative regulator of tyrosine kinase receptor activity. The mRNA level of these genes in GC was correlated negatively with oocyte developmental competence in bovine [65]. Comparison of mRNA patterns after BPA or BPS treatment (20 DEG) confirmed that these two analogues do not activate after 1 h of treatment the same gene regulation mechanisms. Indeed, recent publications have reported a divergence in the mechanisms of action of BPA and BPS [66, 67] and thus corroborate the conclusions from our study. It would therefore be interesting to investigate these genes to characterise their specific mechanisms of action.

### BPA and BPS did not affect the same biological processes

According to GO analysis of DEG, BPA and BPS, although belonging to the same molecular family, exhibited mostly independent early mechanisms of action in ovine GC, highlighted by the low number of common DEG between these conditions compared with the control. Among the common DEG, two genes are involved in apoptosis. *ANKRD1* shows increased expression during oxidative stress in bovine GC [68] and *DDIT4* (also called *REDD1*) is a regulator of caspase-2 (via the ATF4-dependent pathway) in human cancer cells [69].

In this study, BPA rapidly and specifically altered the expression of 200 genes in ovine GC. These genes are mainly related to cellular metabolic processes and regulation of transcription. The protein network of interactions pointed out the proteins coded by *GADD45A* and *GADD45B*. These genes are associated with cell proliferation and survival through the GADD45A–p38–NF-κB and GADD45B–MP2K4–JNK pathways [70], could rapidly modulate signalling in response to physiological and environmental stress. Indeed, BPA may have deleterious effects on the cells by affecting their viability or by causing oxidative stress, as shown in human GC [67]. It would therefore be interesting to decipher GADD45A/GADD45B signalling pathways in ovine GC in response to BPA treatment.

After 1 h of treatment, BPS modulated only a few genes, two being specific to BPS exposure (*RASSL11B* and *RHOB*). Thus, BPS action on the transcription of genes may be delayed compared with BPA. Nevertheless, the GO analysis highlighted cellular communication as the main process altered by BPS. Hence, BPS may affect communication between ovarian follicular cells. In ovine and human thecal cells, BPS increased intercellular gap junction communication, which could lead to folliculogenesis disruption [71]. BPS also affected connexin 37 expression in bovine cumulus cells, therefore altering their communications with enclosed oocyte [66]. Transcriptome analysis did not highlight specific early signalling pathways altered by BPS. This study highlighted the very first genes altered by BPA or BPS exposure. Indeed we expected after only 1 h treatment to study only the direct regulations of BPS, notably due to its binding to receptors, i.e. GPR30. Even though a DEG (*ARRDC4*) related to GPCR endocytosis and pathways was highlighted, it was only after BPA treatment and our pharmacology approach showed that BPA and BPS effects were likely independent from GPR30. To decipher BPS mechanism of action, future studies should analyse the GC transcriptome at a later time of BPS and BPA exposure (6, 12 and 24 h) to determine the full transcriptional changes, even if they are secondary modulations.

### The expression of the LSMEM1 and LIF remained altered by BPA over time

Among the genes upregulated after 1 h of BPA treatment, only *LSMEM1* was still upregulated after 12 h and 24 h of BPA treatment. BPS also upregulated *LSMEM1* early and this gene remained upregulated after 12 and 24 h of BPS treatment. Regarding the expression of *LIF*, downregulated after 1 h of BPA treatment, the decrease in *LIF* expression remained significant up to 48 h of BPA and BPS treatment.

*LSMEM1* and *JUN*, both upregulated by BPA, are linked to the MAPK pathway. *JUN* is a transcription factor, activated in the MAPK signalling pathway, which regulates expression of numerous genes involved in apoptosis, proliferation and cell survival [72, 73]. Activation of this pathway can in particular be promoted by oxidative stress, as shown in human GC [74]. The MAPK signalling pathway was indeed altered by BPA, according to the STRING protein interaction network in the present study. *LSMEM1* can interact with *SRF*, a transcription factor that stimulates the expression of c-fos (belonging to the AP-1 complex with c-jun), and these genes are involved in the control of apoptosis, proliferation and cell differentiation [75]. In addition, a link between the MAPK signalling cascade and the effects of BPA has been reported in diabetic rats, demonstrating alterations in the reno-cardiac axis in response to BPA exposure [76].

*LIF* and *NOTCH1* were downregulated in the presence of BPA in ovine GC after 1 h treatment. *LIF* plays an important role in the regulation of the cell cycle in porcine GC [77]. In addition, *LIF* has recently been shown to modulate the differentiation of ovine GC during terminal folliculogenesis in vitro [78]. *NOTCH1* is a DEG in the BPA versus BPS comparison. In the bovine ovary, the NOTCH signalling pathway is involved in follicular development, steroidogenesis and regression of the corpus luteum [64]. This signalling pathway has already been reported to be impacted in the presence of BPA, disrupting juxtacrine communication in rat testes [79] and steroidogenesis in foetal testes in vitro [80].

Among the most DEG, none encoding key steroidogenesis enzymes were identified after 1 h of treatment with BPA or BPS. Nevertheless, it is interesting to note that several DEG are involved in folliculogenesis. *NR4A1* is an orphan nuclear receptor, able to inhibit oestradiol secretion in human GC [81]. In addition, three members of the family of Kruppel-type transcription factors, SP1 type factors *KLF9*, *KLF10* and *KLF11*, showed a decreased expression in the presence of BPA. Interestingly, they are involved in cell growth, differentiation and apoptosis in ovine and porcine follicular cells [82, 83]. Several genes involved the biosynthesis of steroids in GC, such as *STAR* and *CYP11A*, exhibit Sp1-like sites, which would allow binding with KLF proteins. Thus, inhibition of KLF could increase the transcription of steroidogenesis-related genes, and ultimately the secretions of progesterone and oestradiol, which is opposite to what we observed here. Nevertheless, different KLF are already reported to act through separate pathways and therefore to not have the same effect on steroidogenic actor mRNA [84].

Finally, the genome of the *O. aries* has not yet been fully annotated. Thus, future studies could focus on the 48 DEG that remained to be identified. Indeed, among the genes exhibiting the lowest *p*-value, ENSOARG00000022521 and ENSOARG00000024133, are likely non-coding RNA. It would therefore be interesting to further study bisphenol mechanism of action through non-coding RNA regulations.

## Conclusion

In the present study, we sought to determine in ovine GC, the early mechanisms of action of BPA and BPS on ovine GC, both molecules being already reported to impair steroidogenesis in these cells. While BPS decreased the secretion of both progesterone and oestradiol, BPA only impaired the secretion of progesterone. After treating GC for only 1 h with BPA, BPS or the GPER agonist G-1, RNA-seq analysis suggested that neither BPA nor BPS acted early through GPER. In addition, BPA and BPS exhibited mainly independent mechanisms of action. While BPS had a mild effect on gene expression, BPA altered genes involved in cellular metabolic processes, namely *CHAC1*, *JUN*, *LSMEM1*, *TXNIP* and *ARRDC4*, influencing apoptosis, cellular proliferation and survival.

## Methods

### Chemicals

All chemicals, including BPA and BPS, were obtained from Sigma-Aldrich (Saint-Quentin Fallavier, France), unless otherwise stated in the text. The GPER-specific agonist, G-1 (4-(6-bromo-1,3-benzodioxol-5-yl)-3a,4,5,9b-tetrahydro-3H-cyclopenta[c]quinolin-8-yl] ethanone), and the GPER-specific antagonist, G-15 (4-(6-bromo-1,3-benzodioxol-5-yl)-3a,4,5,9b-3*H*-cyclopenta[*c*]quinolone), were purchased from Tocris (Noyal Châtillon sur Seiche, France). Ethanol was used as a vehicle for BPA, BPS, G-1 and G-15 at the same concentration (1/1111) that was previously described to have no negative impact on the cells and was therefore also at the same concentration in the control condition [33].

### Isolation and culture of GC

GC were recovered from antral follicles (2–6 mm), punctured on 1,000 ovaries of adult ewes from local slaughterhouses, as described previously [33]. Briefly, after cumulus-oocyte complex (COC) depletion, GC were washed in complemented serum-free McCoy’s 5A medium: 3 mM L-glutamine, 0.1% bovine serum albumin, 100,000 UI/L penicillin, 100 mg/L streptomycin, 20 mM HEPES (pH 7.6), 100 nM 4-androsten-11β-ol-3,17-dione (an androgen used by GC as a precursor for oestradiol), 5 mg/L bovine apo-transferrin, 250 nM selenium and 1.72 nM insulin. After GC purification with a Percoll gradient (50% Percoll and 50 M medium), GC were plated at 100,000 viable cells in 150 µL of medium per well in complemented serum-free McCoy’s 5A medium (96-well plates; Thermo Scientific Biolite, Illkirch, France). Depending on the experiment, GC were cultured for 1, 12, 24 or 48 h, with or without BPA or BPS at 10 or 50 µM, or with G-1 at 1 or 10 µM and/or with G-15 at 10 µM. These concentrations were chosen according to previous work regarding the bisphenol concentrations, as these concentrations impaired granulosa steroidogenesis in this model [33] and according to the literature regarding G1 [85] and G15 [86]. BPA and BPS concentrations, which were shown to affect proliferation and/or steroidogenesis of ovine GC in vitro [33], were used in this study. Pre-treatment of the cells with 10 μM G-15 for 1 h was carried out before treatments, followed by treatment with BPA or BPS at 10 or 50 μM. Cultures were performed in a humidified atmosphere with 5% CO_2_ and 20% O_2_ at 38.5 °C.

### Cell viability

Cell viability was assessed after 1 h of treatment in the presence or absence (control) of BPA or BPS at 50 μM, or G-1 at 10 μM (concentrations used for the RNA-seq experiment). GC viability was also assessed after 48 h of treatment in the presence or absence (control) of BPA or BPS at 10 or 50 μM, and/or G-15 at 10 µM or G-1 at 1 μM (concentrations used for steroidogenesis and gene expression studies). Three complementary methods were used to estimate cell viability: Live/Dead staining, the dehydrogenase activity assay (Cell Counting Kit-8 [CCK-8]) and the lactate dehydrogenase activity assay. For Live/Dead staining, GC (250,000 viable cells in 250 µL of complemented serum-free McCoy’s 5A media per well) were cultured on Permanox® Nunc™ Lab-Teck™ eight-Chambers Slide™ (Thermo Fischer Scientific). Live/Dead staining was performed according to the manufacturer’s instructions (Thermo Fischer Scientific). Thus, living cells (green coloured) and dead cells (red coloured) were counted (300 cells minimum per chamber) with ImageJ software (ImageJ-win64). The results are expressed as the percentage of living and dead cells from four and seven independent cultures, at 1 and 48 h of treatment, respectively. Regarding the dehydrogenase activity assay, GC (100,000 viable cells in 150 µL of complemented serum-free McCoy’s 5A media per well) were cultured on 96-well plates (Thermo Scientific Biolite). Cell viability was determined by using the CCK-8, based on the reduction of water-soluble tetrazolium salt (WST-8) to formazan (coloured yellow) by dehydrogenases in living cells, according to the manufacturer’s instructions (Thermo Fischer Scientific). The number of viable cells was determined by colourimetric assay by measuring the absorbance by at 450 nm (Thermolabsystems plate reader, Thermo Fischer Scientific) and Ascent Software version 2.6 for Multiskan. The results are expressed as the mean ± standard error of the mean (SEM) from six and 11 independent cultures at 1 h and 48 h of treatment, respectively. Each condition was performed in triplicate, and the measured values were normalised by the mean value of the control condition. For the lactate dehydrogenase activity assay, GC (100,000 viable cells in 150 µL of complemented serum-free McCoy’s 5A media per well) were cultured on 96-well plates (Thermo Scientific Biolite). The activity of lactate dehydrogenases released by damaged cells was assessed with the Lactate Dehydrogenase Activity Assay Kit, according to the manufacturer’s instructions. This colourimetric assay allowed the determination of the number of dead cells by measuring the absorbance at 450 nm. The results are expressed as the mean ± SEM of 11 independent cultures at 48 h of treatment. Each condition was performed in duplicate, and the measured values were normalised to the mean value of the control condition.

### Progesterone and oestradiol assay

For analyses of steroidogenesis, GC (100,000 viable cells in 150 µL of complemented serum-free McCoy’s 5A media per well) were cultured on 96-well plates. After 48 h of treatment in the presence or absence (control) of BPA or BPS at 10 or 50 μM, and/or G-15 at 10 µM or G-1 at 1 μM, the supernatant and cell layers were separated. Lysis buffer (150 mM NaCl, 10 mM NaF, 12 mM NaH_2_PO_4_, 1 mM Tris, 1 mM EDTA, 2 mM EGTA, 2 mM Na_3_VO_4_, 0.5% NP40 [v/v], 1% Triton X-100 [v/v]) was added to cell layers for protein extraction. The proteins of each well were quantified with the BCA protein quantification kit (Interchim, Montluçon, France). The progesterone and oestradiol concentrations were measured in cell supernatants with enzyme immunoassays. For progesterone, the protocol was described previously [87]; the concentrations ranged from 0.25 to 32 ng/mL, and the intra-assay coefficient of variation (CV) averaged less than 10%. Concentrations of oestradiol were measured according to the manufacturer’s recommendations (E2-EASIA-kit, DIAsource, Louvain-Lan-Neuve, Belgium) and ranged from 1.56 to 50 pg/mL, and the intra-assay CV averaged 15%. Steroid hormone concentrations of each well were normalised by the protein concentrations of the cells from the same well. The results are expressed as the amount of progesterone (ng/mL) or oestradiol (pg/mL) secreted during 48 h per protein amount (µg/mL) per well, as the mean value ± SEM of 10 independent cultures. Each condition was performed in duplicate, and the measured values were normalised to the control value in each culture experiment.

### RNA sequencing and bioinformatics analysis

GC (100,000 viable cells in 150 µL of complemented serum-free McCoy’s 5A media per well) were cultured on 96-well plates. After 1 h of treatment in the presence or absence (control) of BPA or BPS at 50 μM or G-1 at 10 μM, cell layers were collected for RNA extraction using the NucleoSpin® RNA kit (Macherey–Nagel, Hoerdt, France) preceded by DNase treatment. The RNA quantity was assessed with the Qubit® RNA BR Assay Kit (Thermo Fischer Scientific), and the quality of RNA was checked with the Agilent 2100 Bioanalyzer, using the RNA 6000 nano kit (Agilent Technologies, Santa Clara, USA), according to the manufacturers’ recommendations. The samples exhibiting an RNA integrity number (RIN) between 8.9 and 10 (six biological replicates per condition) were included in the RNA-seq experiment. This work has benefited from the facilities and expertise of the high-throughput sequencing core facility of I2BC (Centre de Recherche de Gif – http://www.i2bc.paris-saclay.fr/). From 500 ng of total RNA, directional RNA-seq libraries were created, using the Truseq Stranded Total RNA library prep kit (Illumina). After polyA purification, sequencing was performed on an Illumina NextSeq500 instrument, using the NextSeq 500/550 High Output Kit (75 cycles), to obtain paired-end reads (50–35 nucleotides [nt]). Data analysis was performed with bcl2fastq2-2.18.12 for demultiplexing, Cutadapt 1.15 for adapter trimming and FastQC v0.11.5 for quality control. Reads were mapped on all the genes of the reference genome of sheep (*Ovis aries*, Ensembl version v3.1.103), then counted by using FeatureCounts version 1.6.3.

RNA-seq data were subjected to statistical analysis to identify DEG, using Bioconductor edgeR version 3.24.3. After trimmed mean of M-values (TMM) normalisation to accommodate the different sequencing depths represented by different library sizes, pairwise condition comparisons between all four conditions were executed. Genes with an adjusted *p*-value (padj) ≤ 0.05 (Benjamini–Hochberg correction, to control the false discovery rate [FDR]) were considered statistically significant. The heatmap and principal component analysis were drawn by using XLSTAT (Addinsoft, Paris, France). A hierarchical classification was performed with the mean normalised expression of six biological replicates. For DEG analysis, the effect of inter-replicate variability was taken into account. Raw RNA-seq data were deposited via the SRA Submission portal (https://www.ncbi.nlm.nih.gov/bioproject/PRJNA838746), BioProject ID PRJNA838746.

### Gene ontology (GO) functional analysis

GO enrichment was explored with the R package ViSEAGO [48], with the whole *O. aries* genome (Ensembl version v3.1.103). All enriched GO terms (*p* ≤ 0.01) were grouped into functional clusters using hierarchical clustering based on Wang’s semantic similarity between GO terms by using the GO graph topology and Ward’s criterion. The GO analysis was carried out using three conditions: control, BPA at 50 µM and BPS at 50 μM.

### Real-time polymerase chain reaction (qPCR) and gene expression

Twelve genes, which were reported as differentially expressed by RNA-seq (absolute fold change [|FC|] > 1.5 and padj ≤ 0.05) were also investigated by qPCR in GC through culture kinetics. GC were cultured on 96-well plates (100,000 viable cells in 150 µL of complemented serum-free McCoy’s 5A media per well) for 12, 24 or 48 h in the presence or absence (control) of BPA or BPS at 10 or 50 μM, and/or G-15 at 10 µM. Total RNA was extracted from the cells by using the NucleoSpin® RNA kit (Macherey–Nagel), according to the manufacturer’s instructions. The RNA quantity was determined with a Nanodrop ND-1000 spectrophotometer (Nyxor biotech, Paris, France). DNase treatment and reverse transcription (RT) were performed on 120 ng of total RNA extracted with the Maxima Strand cDNA Synthesis Kit (Thermo Fischer Scientific), according to the manufacturer’s recommendations. qPCR was performed on a CFX384 (Bio-Rad, Marnes-la-Coquette, France) with 125 nM of specific primers (Table 1), RT reaction (diluted 1/15) and qPCR Mastermix Plus for SYBR Green I (Bio-Rad), according to the manufacturer’s instructions. The primer efficiency (E; Table 2) and standard curve were assessed for each gene. GeNorm software was used to confirm that among the genes assessed by qPCR, ribosomal protein L19 (RPL19) and β-actin (ACTB) were the most stable reference genes. All samples underwent qPCR runs for all genes, including RPL19 and ACTB. BioRad CFX Maestro sofware then provide the expression values for each genes (depending on Ct and on the efficiency of the primers). For each sample, the mean of expression values of RPL19 and ACTB is calculated. Then, for each candidate gene, the relative expression level of each sample is divided by the mean expression value of housekeeping genes by using the following equation, where Ct corresponds to the cycle threshold:

**Table 1:** Ovine oligonucleotide primer sequences used in this study

Abbrev	Name	Gene ID	Forward (5ʹ → 3ʹ)	Reverse (5ʹ → 3ʹ)	Size (bp)	E (%)
*ACTB*	β-actine	NM.001009784.3	CCAGCACGATGAAGATCAAG	ACATCTGCTGGAAGGTGGAC	102	109.6
*ARRDC4*	Arrestin domain containing 4	XM_015101774.3	GTTTTGGCAGCAGAAACTCC	GCAAACATAGGACAGCACGA	153	107.7
*CHAC1*	ChaC glutathione specific gamma-glutamylcyclotransferase 1	XM_012181220.4	GTGGTGACCCTCCTTGAAGA	AATGCCTTGAGTGGTTGGTC	182	106.4
*EGR3*	Early growth response 3	XM_027964329.2	ACCTAGCAAGACCCCTCTCC	GCGGATGTGAGTGGTAAGGT	187	102.4
*JUN*	Jun proto-oncogene, AP-1 transcription factor subunit	XM_004002020.5	ACGACCTTCTACGACGATGC	TGAGGAGGTCGGAGTTCTTG	166	106.8
*KLF9*	Kruppel like factor 9	XM_042243358.1	CCCAGTGTCTGGTTTCCATT	CTGGATGGGTCGGTACTTGT	197	106.2
*KLF10*	Kruppel like factor 10	XM_027973155.2	CCTTCCTCAGCGAAAGTCAC	CAGGCGAACTTCTTCTCACC	238	98.9
*KLF11*	Kruppel like factor 11	XM_004005679.5	TCATTGCATCCAGTCAGAGC	TGCAGCTGAAAGGCTTCTCT	156	105.8
*LIF*	LIF interleukin 6 family cytokine	XM_042234817.1	TGGAGCTGTATCGCATCATC	TGACAGCCCAGCTTCTTCTT	250	100.7
*LSMEM1*	Leucine Rich Single-Pass Membrane Protein 1	XM_042249200.1	GAAACGTTCTTCCCAGGACA	ATCACGAAGGAAACCAGTGC	243	91.2
*NOTCH1*	Notch Receptor 1	XM_027966164.2	CGATGAGTGTGAGTCGAACC	AGGCAGTTGCATTTGTACCC	192	102.4
*NR4A1*	Nuclear receptor subfamily 4 group A member 1	XM_042246696.1	GGGGGAGGGAGAGAGCTATT	ACCATAATGCTGGCAGGAAG	187	103.1
*RPL19*	Ribosomal protein L19	AY158223.1	CACAAGCTGAAGGCAGACAA	TGATGATTTCCTCCTTCTTGG	130	103.9
*TXNIP*	Thioredoxin interacting protein	GAAI01004920.1	GCCTCTGGGAACATCTTTCA	TTCGTGCAGAGACAGACACC	248	99.4

$$\mathrm R=\frac{(E_{gene}^{-Ctgene})}{(\mathrm{geometric}\;\mathrm{mean}\left(E_{ACTB}^{-CtACTB};E_{RPL19}^{-CtRPL19}\right))}.$$

The relative abundance of transcripts (R) are presented as the mean ± SEM of eight independent cultures, normalised to the control value at 12 h.

### Statistical analysis

Statistical analyses were performed with R version 4.1.0 using the R commander package [88]. Cell viability (except Live/Dead staining), steroidogenesis and gene expression were analysed with non-parametric analysis of variance (ANOVA) by permutation (lmperm package). The treatment effect, culture effect and treatment-by-culture interactions were assessed. Tukey’s post hoc test (nparcomp package) was performed to determine differences between conditions. Logistic regression analysis was used to analyse Live/Dead staining. Significant difference was indicated when *p* ≤ 0.05, and a tendency when 0.05 < *p* ≤ 0.1.

## Supplementary Information

**Additional file 1: Supplemental Figure S1.** The effects of the GPER-specific agonist (G-1) or antagonist (G-15) and/or bisphenol A (BPA) and bisphenol (BPS) on ovine granulosa cells (GC) cell viability. **Supplemental Figure S2.** Functional analysis of differentially expressed genes (DEG) from RNA-sequencing of ovine granulosa cells (GC) treated with bisphenol A (BPA) or bisphenol S (BPS). **Supplemental Figure 3.** Interactions between of proteins encoded by differentially expressed genes (DEG) after treatment with bisphenol A (BPA), using STRING.

**Additional file 2: Supplementary Table 1.** Comparisons of the effect on progesterone (**A**) and oestradiol (**B**) secretion between conditions, including the *p*-values for all the multiple comparisons of the post-hoc test that followed the ANOVA. * indicates a significant difference and # indicates a tendency. **Supplementary Table 2.** Total number of reads for each sample. Six biological replicates of ovine granulosa cells were sequences per experimental conditions (Control, 50 µM BPA, 50 µM BPS, 10 µM G1), after 1h of treatment. This analysis generated an average of 23.7 ± 0.5 million reads per sample, and 73.7 ± 0.2 % of the reads were uniquely aligned with the sheep reference genome (Oar_rambouillet_v1.0, chromosomes). **Supplementary Table 3.** Differentially expressed genes (adjusted *p*-value ≤ 0.05) between the four comparisons: control versus bisphenol A (BPA), control versus bisphenol S (BPS), BPA versus BPS and G1 versus control. Six biological replicates of ovine granulosa cells were sequenced per experimental conditions (Control, 50 µM BPA, 50 µM BPS, 10 µM G1), after 1h of treatment. **Supplementary Table 4.** Hierarchical list of differentially expressed genes in the heatmap presented in Figure 2B. **Supplementary Table 5.** Functional analysis of ovine granulosa cells differentially expressed genes. Functional clusters using hierarchical clustering based on Wang’s semantic similarity between GO terms respecting GO and Ward’s criterion were performed using the ViSEAGO package. **Supplementary Table 6.** The main biological processes of differentially expressed genes (DEG) after treatment with bisphenol A (BPA) or bisphenol S (BPS). After 1 hour of treatment, in the presence or absence (control) of BPA or BPS at 50 μM, GC (6 replicates / condition) were an RNA-seq analysis made it possible to obtain a list of differential expression genes. Biological processes from gene ontology (GO; *p* ≤ 0.01) terms was obtained using ViSEAGO. The table shows the 20 GO, corresponding to biological functions, the most disturbed by BPA or BPS. The logarithmic threshold *p*-value was set at 2, corresponding to a *p*-value of 0.01. **Supplementary Table 7.** **A** Comparison of gene expression at a given culture time between treatments. Gene expression is presented as mean ± SEM and normalized to the control at 12 h (different letters indicate a significant difference between treatments).

## Data Availability

The Raw RNA-seq datasets generated and/or analysed during the current study are available in the via the SRA Submission portal, (https://www.ncbi.nlm.nih.gov/bioproject/PRJNA838746), BioProject ID PRJNA838746. All other data generated or analysed during this study are included in this published article [and its supplementary information files].
