# Supplementary material for: Bisphenol A and bisphenol S both disrupt ovine granulosa cell steroidogenesis but through different molecular pathways
Source: J Ovarian Res. 2023 Feb 3;16:30. doi: 10.1186/s13048-023-01114-4 (PMC9896735; doi:10.1186/s13048-023-01114-4)
Supplement: Supplementary file 1 — Additional file 1: Supplemental Figure S1. The effects of the GPER-specific agonist (G-1) or antagonist (G-15) and/or bisphenol A (BPA) and bisphenol (BPS) on ovine granulosa cells (GC) cell viability. Supplemental Figure S2. Functional analysis of differentially expressed genes (DEG) from RNA-sequencing of ovine granulosa cells (GC) treated with bisphenol A (BPA) or bisphenol S (BPS). Supplemental Figure 3. Interactions between of proteins encoded by differentially expressed genes (DEG) after treatment with bisphenol A (BPA), using STRING. [file 13048_2023_1114_MOESM1_ESM.pdf]

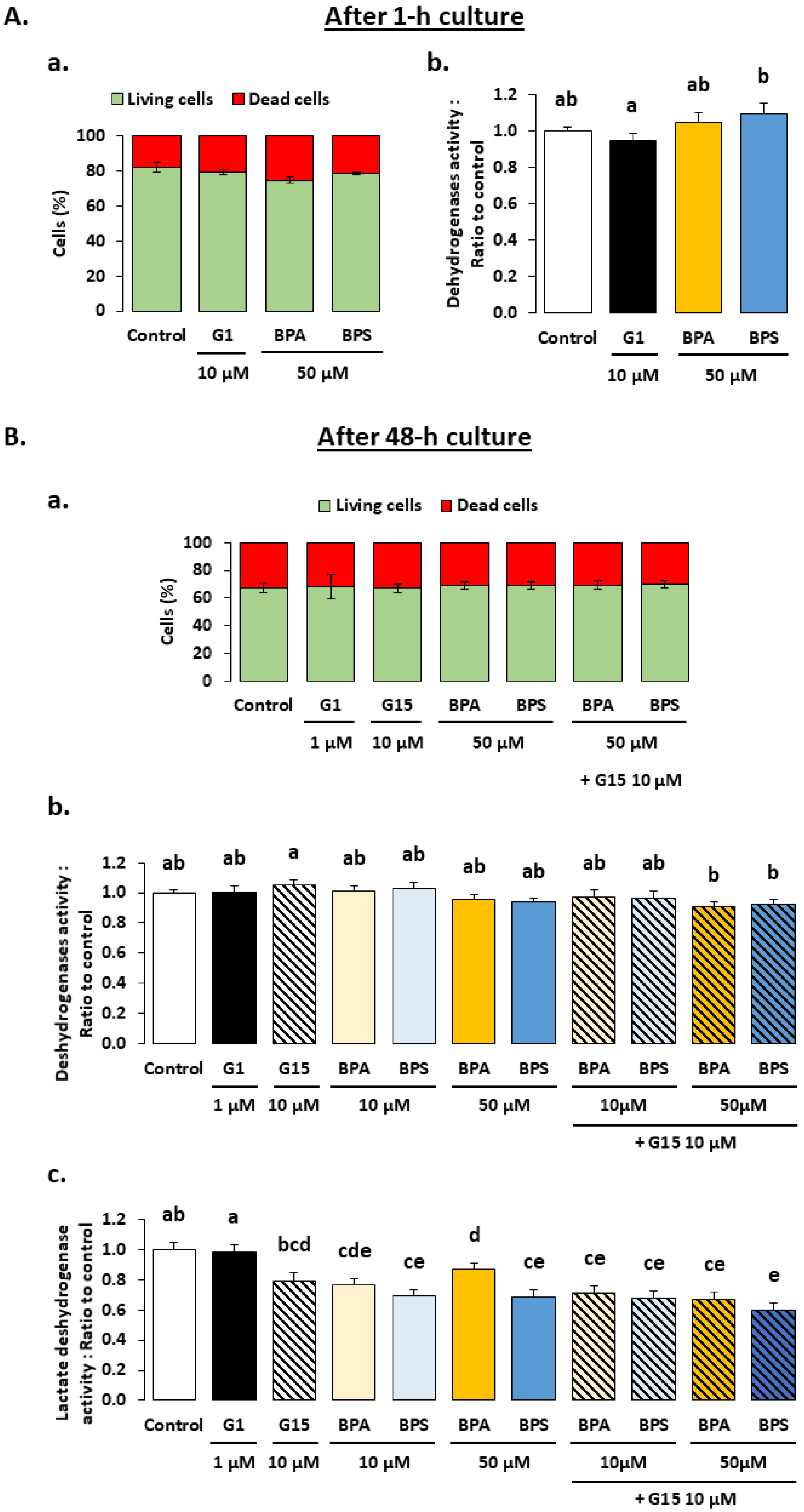
A.
After 1-h culture
a.
Living cells
Dead cells
Cells (%)
100
80
60
40
20
0
Control
G1
BPA
BPS
10 µM
50 µM
b.
Dehydrogenases activity :
Ratio to control
1.2
1.0
0.8
0.6
0.4
0.2
0.0
ab
a
ab
b
Control
G1
BPA
BPS
10 µM
50 µM
B.
After 48-h culture
a.
Living cells
Dead cells
Cells (%)
100
80
60
40
20
0
Control
G1
G15
BPA
BPS
BPA
BPS
1 µM
10 µM
50 µM
50 µM
+ G15 10 µM
b.
Deshydrogenases activity :
Ratio to control
1.2
1.0
0.8
0.6
0.4
0.2
0.0
ab
ab
a
ab
ab
ab
ab
ab
ab
b
b
Control
G1
G15
BPA
BPS
BPA
BPS
BPA
BPS
BPA
BPS
1 µM
10 µM
10 µM
50 µM
10µM
50µM
+ G15 10 µM
c.
Lactate deshydrogenase
activity : Ratio to control
1.2
1.0
0.8
0.6
0.4
0.2
0.0
ab
a
bcd
cde
ce
d
ce
ce
ce
ce
e
Control
G1
G15
BPA
BPS
BPA
BPS
BPA
BPS
BPA
BPS
1 µM
10 µM
10 µM
50 µM
10µM
50µM
+ G15 10 µM

Supplemental Figure S1: The effects of the GPER-specific agonist (G-1) or antagonist (G-15) and/or bisphenol A (BPA) and bisphenol (BPS) on ovine granulosa cells (GC) cell viability.

Cell viability was checked after 1 h (A) and 48 h (B) culture in complemented serum-free McCoy's 5A media. Cell viability was assessed after 1 h (A) of treatment in the presence or absence (control) of BPA or BPS at 50 µM or G-1 at 10 µM (used for the RNA-sequencing experiment), with two complementary methods: Live/Dead staining (A.a) and the dehydrogenase activity assay (A.b). For Live/Dead staining (A.a), the results are expressed as the percentage of four independent cultures. For the dehydrogenase activity assay (A.b), the results are expressed as the mean ± standard error of the mean (SEM) of six independent cultures. Each condition was performed in duplicate and normalised to the control condition of each culture experiment. Bars with different superscripts indicate a significant difference ($p \leq 0.05$). Cell viability was assessed after 48 h (B) of treatment (maximum cell culture time in this paper) in the presence or absence (control) of BPA or BPS at 10 or 50 µM, and/or G-15 at 10 µM or G-1 at 1 µM (used for steroidogenic activity and genes expression), with three complementary methods: Live/Dead staining (B.a), the dehydrogenase activity assay (B.b) and the lactate dehydrogenase activity assay (B.c). For Live/Dead staining (B.a), the results are expressed as the percentage of seven independent cultures. For the dehydrogenase activity assay (B.b) and the lactate dehydrogenase activity assay (B.c), the results are expressed as the mean ± SEM of 11 independent cultures. Each condition was performed in duplicate and normalised to the control condition of each culture experiment. Bars with different superscripts indicate a significant difference ($p \leq 0.05$).

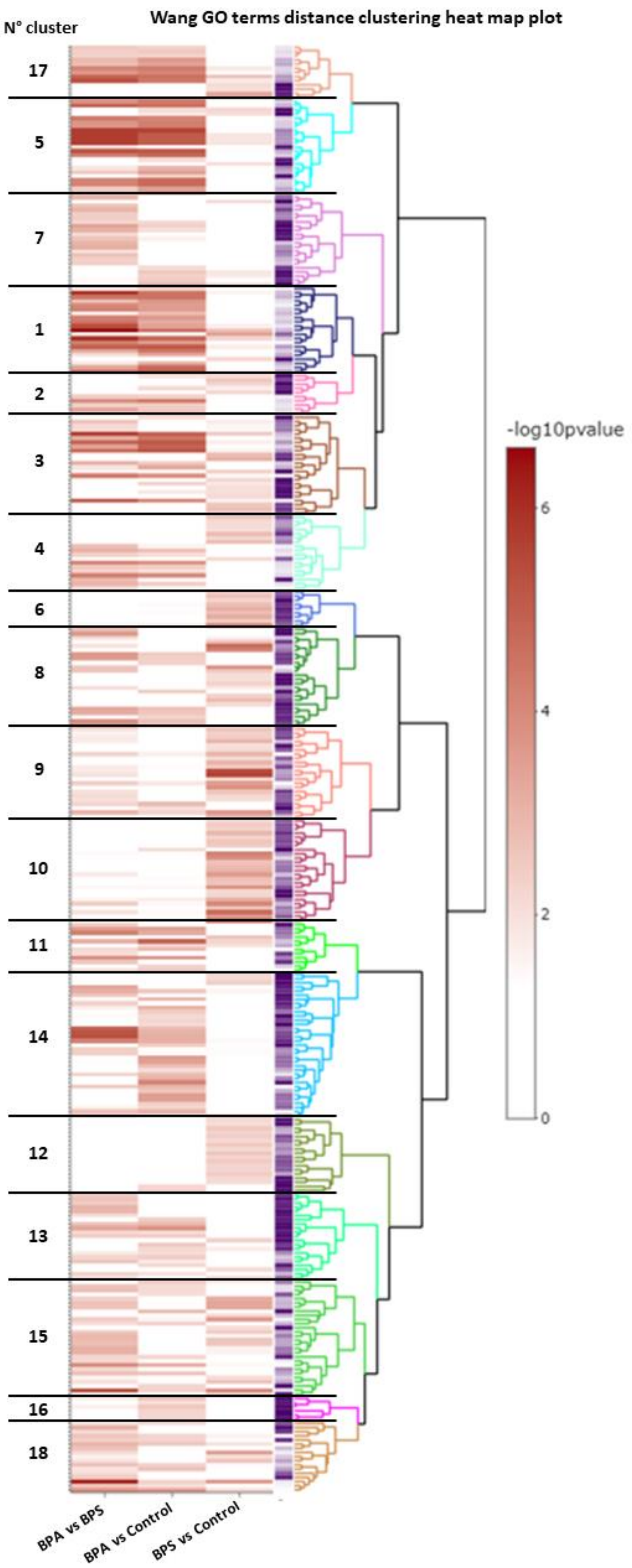
Wang GO terms distance clustering heat map plot
N° cluster
17
5
7
1
2
3
4
6
8
9
10
11
14
12
13
15
16
18
BPA vs BPS
BPA vs Control
BPS vs Control
-log10pvalue
6
4
2
0

Supplemental Figure S2: Functional analysis of differentially expressed genes (DEG) from RNA-sequencing of ovine granulosa cells (GC) treated with bisphenol A (BPA) or bisphenol S (BPS).

After 1 h of treatment, in the presence or absence (control) of BPA or BPS at 50 μM, GC (six replicates per condition) were analysed with RNA-sequencing to obtain a list of DEG ($p \leq 0.05$). The clustering heatmap plot for each condition with pairwise comparisons (BPA vs BPS, BPA vs control, BPS vs control) of functional sets of gene ontology (GO) terms ($p \leq 0.01$) was obtained by using ViSEAGO. From left to right are: the cluster number, the heatmap of -log10 (p-value) of GO terms from functional enrichment tests, the information content (IC) and the dendrogram based on Wang's semantic similarity distance and Ward's clustering criterion. The list with GO terms corresponding to the heatmap have been annotated in Supplemental Table S4.

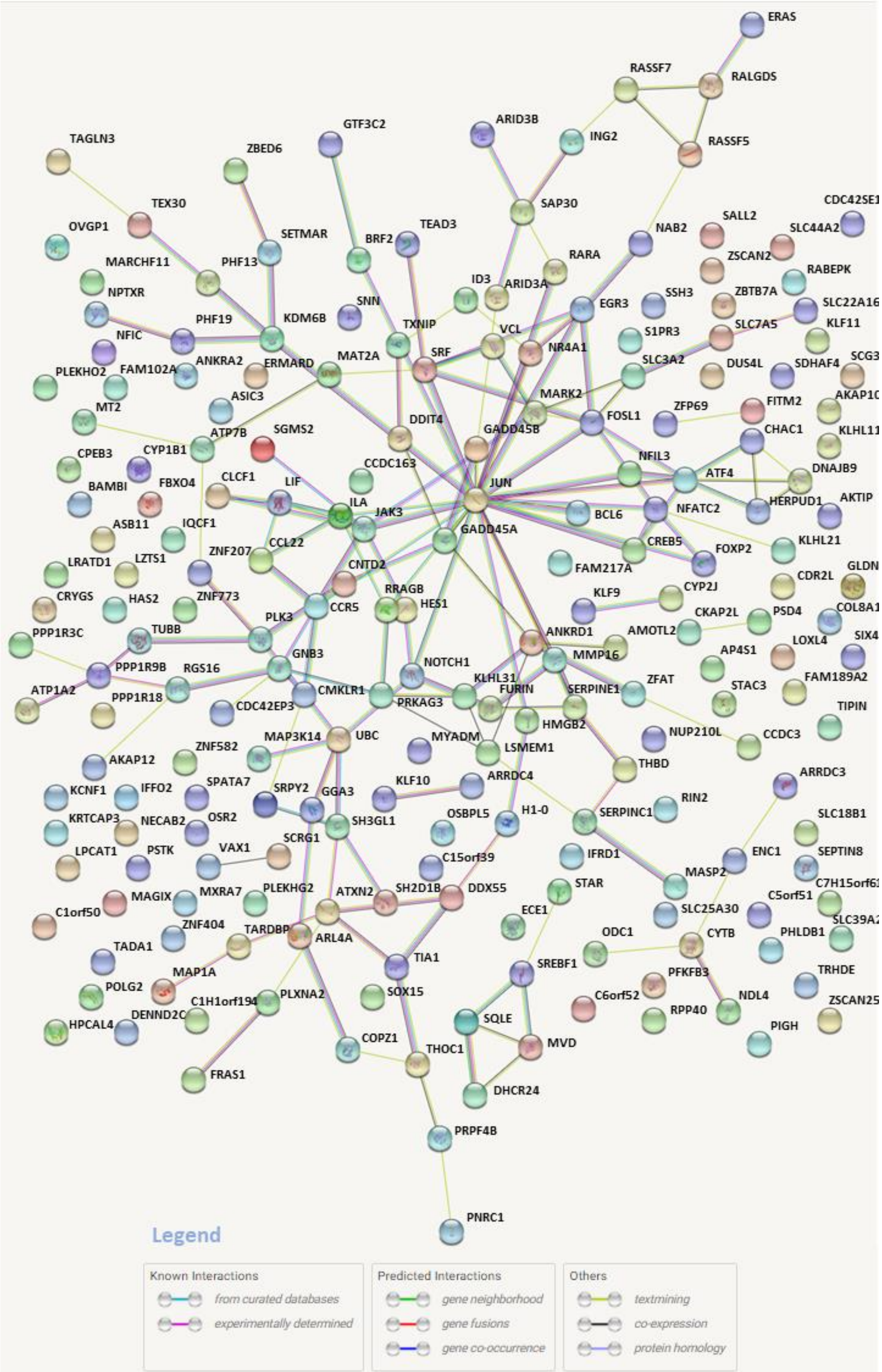

DATABASE KNOWLEDGE

Relevant datasets in Ovis aries:

annotated pathway (KEGG)<br>*Name: Metabolic pathways*	ND4L MVD SQLE MAT2A CYTB ODC1 SETMAR SGMS2 CYP2J PIGH DHCR24 CHAC1 [... and 1251 other proteins]
annotated pathway (KEGG)<br>*Name: MAPK signaling pathway*	SRF ATF4 IL1A MAP3K14 GADD45A NR4A1 GADD45B [... and 216 other proteins]

Supplemental Figure 3: Interactions between of proteins encoded by differentially expressed genes (DEG) after treatment with bisphenol A (BPA), using STRING

After 1 h of treatment, in the presence or absence (control) of BPA at 50 µM, GC (six replicates per condition) were analysed with RNA-sequencing to obtain a list of 259 DEG ($p \leq 0.05$), of which 214 had been identified. Interactions between proteins from these 214 genes and the most represented signalling pathways were assessed with STRING version 11.5 (http://string-db.org/). The intensity of the edges reflects the strength of the interaction score.
